# Continuous peritoneal lavage with vacuum peritoneostomy: an experimental study

**DOI:** 10.6061/clinics/2019/e937

**Published:** 2019-07-03

**Authors:** Adilson Costa Rodrigues, Karen Ruggeri Saad, Paulo Fernandes Saad, Denise Aya Otsuki, Luana Carla dos Santos, Samir Rasslan, Edna Frasson de Souza Montero, Edivaldo M Utiyama

**Affiliations:** I Cirurgia, Hospital das Clinicas HCFMUSP, Faculdade de Medicina, Universidade de Sao Paulo, Sao Paulo, SP, BR; II Faculdade de Medicina, Universidade Federal do Vale do São Francisco, Petrolina, PE, BR; III Laboratorio de Anestesiologia (LIM-08), Faculdade de Medicina FMUSP, Universidade de Sao Paulo, Sao Paulo, SP, BR; IV Laboratorio de Fisiopatologia Cirurgica (LIM-62), Faculdade de Medicina FMUSP, Universidade de Sao Paulo, Sao Paulo, SP, BR; V Clinica Cirurgica III, Hospital das Clinicas HCFMUSP, Faculdade de Medicina, Universidade de Sao Paulo, Sao Paulo, SP, BR

**Keywords:** Peritonitis, Open Abdomen, Laparostomy, Peritoneal Lavage, Vacuum-Assisted Closure, Abdominal Sepsis

## Abstract

**OBJECTIVE:**

Despite advances in diffuse peritonitis treatment protocols, some cases develop unfavorably. With the advent of vacuum therapy, the use of laparostomy to treat peritonitis has gained traction. Another treatment modality is continuous peritoneal lavage. However, maintaining this technique is difficult and has been associated with controversial results. We propose a new model of continuous peritoneal lavage that takes advantage of the features and benefits of vacuum laparostomy.

**METHOD:**

Pigs (Landrace and Large White) under general anesthesia were submitted to laparostomy through which a multiperforated tube was placed along each flank and exteriorized in the left and lower right quadrants. A vacuum dressing was applied, and intermittent negative pressure was maintained. Peritoneal dialysis solution (PDS) was then infused through the tubes for 36 hours. The stability of peritoneostomy with intermittent infusion of fluids, the system resistance to obstruction and leakage, water balance, hemodynamic and biochemical parameters were evaluated. Fluid disposition in the abdominal cavity was analyzed through CT.

**RESULTS:**

Even when negative pressure was not applied, the dressing maintained the integrity of the system, and there were no leaks or blockage of the catheters during the procedure. The aspirated volume by vacuum laparostomy was similar to the infused volume (9073.5±1496.35 mL *versus* 10165±235.73 mL, *p*=0.25), and there were no major changes in hemodynamic or biochemical analysis. According to CT images, 60 ml/kg PDS was sufficient to occupy all intra-abdominal spaces.

**CONCLUSION:**

Continuous peritoneal lavage with negative pressure proved to be technically possible and may be an option in the treatment of diffuse peritonitis.

## INTRODUCTION

Indications for the strategy known as the open abdomen technique (OAT) include the treatment of severe abdominal sepsis, the prevention or treatment of intra-abdominal hypertension, and the need for damage control in cases of intra-abdominal bleeding 1. However, the treatment of diffuse peritonitis, despite advances, remains challenging 2,3.

It is known that temporary closure of the abdomen with the use of traditional dressings is associated with increased mortality and morbidity compared with closure of the abdomen and relaparotomy on demand 4-7. However, a recent study suggested that the OAT with closure of the abdomen using dressings that provide negative pressure is associated with favorable results 1.

With the advent of vacuum therapy, the use of laparostomy to treat diffuse peritonitis has gained traction 1,8. Despite these advances, patients treated with OAT are submitted to many procedures, and extensive time is generally spent in the intensive care unit because, regardless of the technique employed, the laparostomy device must be changed several times until the patient recovers.

Another modality of treatment of diffuse peritonitis is continuous peritoneal lavage 9, which aims to decrease the number of procedures and maintain continuous washing concurrently with intensive care support. However, the concept of continuous peritoneal lavage is technically difficult and has been associated with controversial results 3.

No technique is currently available that reduces the number of procedures and the stress of dressing removal in OAT. Additionally, no technique combines the benefits of vacuum laparostomy with continuous peritoneal lavage. The concept of irrigation and aspiration through the vacuum system is currently used only for superficial wounds 10,11.

Thus, in this experiment, we evaluated the performance of a model of continuous peritoneal lavage taking advantage of the features and benefits of vacuum laparostomy, and we analyzed the viability of continuously maintaining fluid infusion in the peritoneum through an intermittent vacuum laparostomy dressing.

## MATERIAL AND METHODS

### Study design and ethical statement

The research protocol was approved by the Research Ethics Committee for the use of animal models at our institution (Protocol No. 086/12). All animals were handled according to the principles of the National Institutes of Health (1985) and The American Physiological Society (1995) for the care, handling and use of laboratory animals. An experimental study was carried out in which four Landrace and Large White swine weighing between 20 and 30 kg underwent the experimental procedure. Exclusion criteria or animals were a plasma hemoglobin concentration lower than 9 mg/dL, abnormal blood gas values at baseline, clinical signs of infection, and early hemodynamic deterioration (blood loss <500 mL associated with mean arterial pressure (MAP) <60 mmHg in the first 10 minutes. After the experimental procedure, the animals were sacrificed with an overdose injection of sodium phenobarbital.

### Anesthetic protocol, instrumentation and monitoring

The preparation of the animals included fasting for 12 hours with access to water ad libitum. Then, the animals were sedated with an intramuscular injection of ketamine (5 mg kg ^-1^) and midazolam (0.25 mg kg ^-1^), and anesthesia was induced with i.v. administration of propofol (5 mg kg ^-1^).

After endotracheal intubation, anesthesia was maintained with isoflurane (1.5% by volume), and pancuronium bromide (5 mg kg^-1^ min^-1^) was used to ensure muscle paralysis, which was monitored throughout the experiment.

The ventilation was adjusted for volume control of the following parameters: FiO2 of 40%, 8 mL kg^-1^, 5 cmH2O of positive end-expiratory pressure (PEEP) and a respiratory rate adequate to maintain PaCO_2_ between 30 mmHg and 35 mmHg. A heating blanket was used to maintain the temperature of the animals at approximately 38°C.

A multiparametric monitor (IntelliVue MP40; Phillips, Boeblinger, Germany) was used to evaluate heart rate (HR) and rhythm throughout the procedure.

The right internal jugular vein was punctured for the introduction of a continuous pulmonary artery catheter coupled with a mixed oxygen venous saturation monitor (SvO2) connected to a cardiac output monitor. A catheter was introduced via the pressure transducer connection and was guided by analyzing the pressure curves of AD, VD, AP and POAP. The midpoint between the anterior and posterior thoracic walls was taken as the zero reference point for pressure measurements. The intravascular catheters were adjusted to the atmospheric parameters.

A standard Foley catheter was positioned in the bladder and connected to a pressure transducer for assessment of intra-abdominal pressure (IAP).

### Technique

The animals were submitted to a continuous peritoneal lavage technique associated with vacuum peritoneostomy, as previously described 12. They were submitted to laparostomy through which a 4.8 mm multiperforated tube was placed along each flank and exteriorized in the lower left and lower right quadrants. An adapted vacuum dressing was placed in the laparostomy (VAC® Dressing, KCI), and an intermittent negative pressure of 125 mmHg was maintained with vacuuming every 30 minutes for 30 minutes.

Hemodynamic and biochemical parameters and bladder pressure values were collected at baseline (BL), 6 hours (T6), 12 hours (T12), 18 hours (T18), 24 hours (T24) and 36 hours (T36).

### CT image evaluation

The animals received 60 ml/kg PDS through the two multiperforated tubes and were submitted to CT without the use of intravenous contrast.

To quantify the fluid present in the cavity, a scoring system called the abdominal fluid distribution score (AFDS) was used. The AFDS assesses the presence or absence of fluid and the amount of fluid visualized on CT images. The abdominal cavity of the animal was divided into 6 regions: the right costophrenic recess (RCFR), the left costophrenic recess (LCFR), the subhepatic region (SH), between loops (BL), the right iliac fossa (RIF) and the left iliac fossa (LIF).

For AFDS analysis, one point was assigned for liquid presence, and two points were assigned if the presence of liquid was greater than 1 cm in the image for each region. The minimum score was zero, i.e., no fluid in any space, and the maximum score was twelve corresponding to the presence of fluid in all spaces with a thickness greater than 1 cm in the CT image. A score of 6 was considered sufficient to perform washing in this model.

### System stability and physiological effects of washing with PDS

Peritoneostomy stability with intermittent infusion of fluids, system resistance to obstructions and leakage, water balance, hemodynamic parameters (mean arterial pressure, HR, IAP, diuresis, CO) and biochemical parameters (pH, lactate, sodium, potassium, hematocrit and SatO) were analyzed every 6 hours.

### Statistical analysis

Data were analyzed using the SigmaStat program for Windows and are presented as medians. The Wilcoxon paired test was used to compare hemodynamic and biochemical variables collected at the beginning and end of the experiment.

## RESULTS

### Disposition of the peritoneal lavage fluid in the abdominal cavity

Through analysis of CT images, it was possible to verify efficiency of the system in occupying all spaces. Table 1 shows the scoring results using the AFDS. The results were considered adequate according to established criteria.

**Figure 1:**
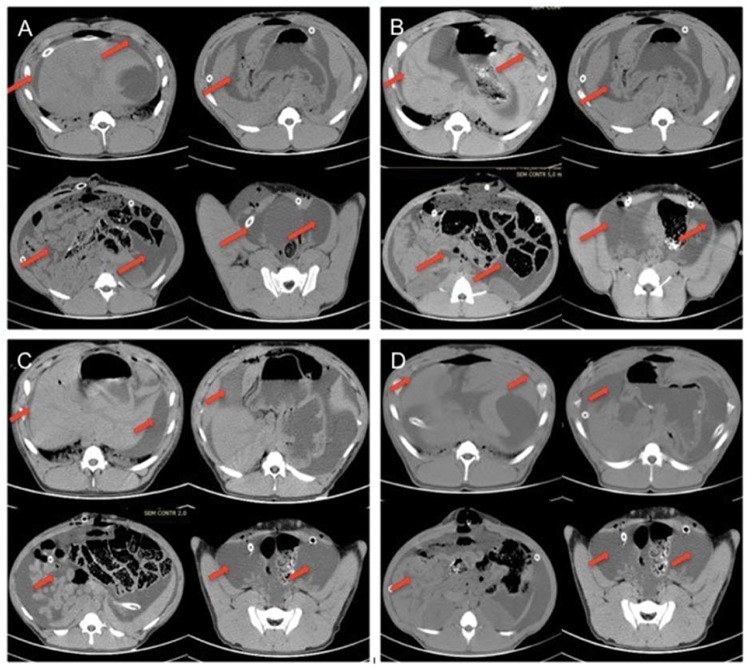
(A) Pig 1, (B) Pig 2, (C) Pig 3 and (D) Pig 4. The red arrows indicate the presence of liquid in all spaces evaluated.

Although previously positioned in the right and left flanks, the multiperforated tubes left their original position in all animals. However, this movement of the tubes did not affect the distribution of fluids within the abdominal cavity (Figures 1(A-D)).

### Stability and physiological effects

Stability of the peritoneostomy was maintained during the procedure, with no leakage, catheter obstruction or changes in IAP. The water balance evaluation showed that the volume aspirated by peritoneostomy at the end of the experiment was similar to the volume infused (10165±235.73 mL *versus* 9073.5±1496.35 mL, *p*=0.25). There were no significant changes in hemodynamic or biochemical parameters (Tables 2 and 3).

## DISCUSSION

The continuous peritoneal lavage strategy as a treatment for abdominal sepsis is supported by the idea that continuous irrigation of the abdominal cavity washes out inflammatory mediators and contaminants, thus resolving the infection.

This theory has been studied since the 1960s, although there is little evidence supporting this approach in clinical and scientific literature 13,14. It has also been postulated that current techniques and more aggressive washing may cause more harm than good 9.

Clinical and experimental controversial and unfavorable results of continuous peritoneal lavage may be associated with the approaches that are currently used. These approaches are not capable of washing the entire cavity and/or do not effectively clear contaminants and inflammatory mediators, since they do not completely irrigate the abdominal cavity. Intracavitary catheters are generally positioned, and it is expected that irrigation, without proper removal of the infused fluid, will resolve the intra-abdominal infection. In addition, the abdominal cavity is kept closed, which contributes to increased abdominal pressure. A few studies have evaluated peritoneostomy with peritoneal lavage, but there are no comparative studies 15,16.

Finally, the technical difficulties of continuous peritoneal lavage are associated with maintaining the integrity of the system. Most methods use the same fluid infusion route to aspirate the lavage, causing leaks, obstructions, contamination and infection of the remaining volume 3.

In a recent publication 12, we described a continuous peritoneal lavage model associated with vacuum peritoneostomy that aimed to solve the problems observed in previous peritoneal lavage techniques. The model was able to maintain stability of the lavage system for 9 hours without leaks or obstruction and resulted in adequate clearance. However, the mean volume of saline that was aspirated by peritoneostomy during the experiment was greater than the volume that was infused by the catheters. In addition, the animals presented hemodynamic and biochemical changes at the end of the experiment, which was attributed to the lavage solution.

Previous continuous peritoneal lavage techniques also used saline solution as the lavage fluid. However, the literature suggests that the use of 0.9% saline solution may be prejudicial to the peritoneal mesothelium 17,18 and cause hydroelectrolytic disturbances 3. Thus, in this study, we chose to use PDS as a lavage solution, which, besides being less harmful to the mesothelium, also has the advantage of assisting in the clearance of contaminants and inflammatory products resulting from sepsis. The results presented with prolonged washing with PDS showed that PDS use does not interfere with the homeostasis of the animal, suggesting that PDS is the best solution for maintaining continuous peritoneal lavage.

Regarding the lavage capacity of the system, the analysis of CT images suggested the potential of this technique to reach the entire abdominal cavity. In addition, the use of multiperforated tubes for infusion and the fluid collected by peritoneostomy allowed all the infused contents to be recovered, even though a large volume of liquid per wash (60 ml/kg) was used.

Regarding maintenance of the integrity of the system, the results of this study showed that the technique promoted stability, without obstructions or leaks for 36 hours. The association between peritoneostomy and continuous peritoneal lavage maintains system stability by preventing leakage. In addition, the open abdomen allows for greater cavity compliance and normal IAP values, despite the volume added to the cavity during lavage. Methods that maintain the cavity closed with suturing of the aponeurosis lead to increased IAP and the development of abdominal compartment syndrome 3,19.

Clinical studies should be performed to evaluate whether continuous peritoneal lavage with PDS associated with vacuum peritoneostomy may be useful in the treatment of diffuse peritonitis.

## CONCLUSION

Continuous peritoneal lavage with PDS associated with vacuum peritoneostomy is technically feasible and maintains physiological parameters within the normal range. Thus, clinical studies should be conducted to evaluate the use of this technique in the treatment of diffuse peritonitis.

## AUTHOR CONTRIBUTIONS

Rodrigues AC and Utiyama EM were responsible for the conception, design, intellectual and scientific content of the study. Santos LC and Otsuki DA were responsible for animal procedures, management, monitoring and data collection. Saad KR and Saad PF were responsible for statistics and manuscript writing. Montero EFS, Rasslan S, Utiyama EM were responsible for interpretation of the data, discussion and ensuring adequacy of the model for clinical applications. All authors revised the final text of the manuscript and contributed to the final version.

## Figures and Tables

**Table 1:** Abdominal Fluid Distribution Score assessed from CT images.

Animals	RCFR	LCFR	SH	BL	RIF	LIF	Total
1	1	2	2	1	2	2	**10**
2	1	1	2	1	2	2	**9**
3	1	1	2	2	2	2	**10**
4	1	1	2	1	2	2	**9**

RCFR: Right costophrenic recess; LCFR: Left costophrenic recess; SH: Subhepatic; BL: Between loops; RIF: Right iliac fossa; LIF: Left iliac fossa.

**Table 2:** Hemodynamic variables of animals submitted to peritoneal lavage with PDS.

	BL (median)	T6 (median)	T12 (median)	T18 (median)	T24 (median)	T36 (median)	*p*-value*
MAP (mmHg)	66.5	64.5	62.5	61.5	68.5	65	0.42
HR (bpm)	91.5	101	87.5	106	103	97.5	0.71
IAP (mmHg)	5.5	5.5	8	8	17.5	13	0.07
Diuresis (mL)	350	280	442	566	642	900	0.07
CO (L/min)	2.7	3	2.65	2.8	2.45	2.95	0.27

PDS: Peritoneal dialysis solution; MAP: Mean blood pressure; HR: Heart rate; IAP: intra-abdominal pressure; CO: Cardiac output; * Paired Wilcoxon test (T0XT36).

**Table 3:** Biochemical variables of animals submitted to peritoneal lavage with PDS.

	BL (median)	T6 (median)	T12 (median)	T18 (median)	T24 (median)	T36 (median)	*p*-value*
pH	7.44	7.49	7.49	7.49	7.48	7.47	0.14
Lactate (nmol/L)	1.15	0.82	1.07	1.02	0.77	0.74	0.14
Na (nmol/L)	138.50	133.5	136	134	133.50	134.50	0.07
K (nmol/L)	3.77	4.56	4.25	4.21	4.05	4.31	0.07
Ht (%)	27.40	30.60	29.95	29.70	33.85	29.90	0.59
SatO_2_ (%)	99.50	99.80	99.40	99.50	99.50	97.95	0.99

PDS: Peritoneal dialysis solution; pH: Hydrogen ionic potential; Na: Serum sodium; K: Serum potassium; Ht: Hematocrit; SatO2: arterial oxygen saturation; * Paired Wilcoxon test (T0XT36).
